# A Multidimensional Approach to Measure Structural Racism and Its Association with Fatal and Nonfatal Community Firearm Violence at the Census Tract Level in New York City

**DOI:** 10.1007/s11524-025-01001-x

**Published:** 2026-06-22

**Authors:** Loren L. Adams, Rashelle J. Musci, Vanya Jones, Cassandra K. Crifasi

**Affiliations:** Johns Hopkins University Bloomberg School of Public Health, Baltimore, MD USA

**Keywords:** Structural racism, Latent profile analysis, Health equity, Community firearm violence

## Abstract

Structural racism is an important determinant of health inequities. Recent studies have operationalized structural racism using latent construct approaches to capture its multidimensionality instead of relying on single indicators as structural racism proxies. However, such studies have only captured multidimensional structural racism at larger geographies, not census tract level. A latent profile model used unidimensional indicators across the domains of criminal justice, economic status, education, employment, and housing/segregation to construct a multidimensional measure of structural racism (MMSR) for 2219 NYC census tracts from 2018–2022. The indicators were derived from publicly available datasets and reflect differences between Black and non-Hispanic White New Yorkers. We assessed the MMSR’s utility by testing its association with fatal and nonfatal community firearm violence in 2018–2022, using a global Wald test and pairwise comparison z-tests. The analysis identified five multidimensional structural racism profiles in NYC. Two profiles were more advantaged across the indicators, while the other three were more disadvantaged. Census tracts in the advantaged profiles had above the NYC average of non-Hispanic White residents and above median income, while disadvantaged profiles had above average non-Hispanic Black residents and below median income. Community firearm violence significantly differed across the five profiles—the disadvantaged profiles had the highest prevalence of firearm violence. Maps visualized that disadvantaged profiles co-occurred in census tracts that also experience high levels of violence. This analysis confirms the need to capture the complexity of structural racism across multiple domains, including the heterogeneity of structural racism at a small geography in a large city.

**Supplementary Information:**

The online version contains supplementary material available at https://doi.org/10.1007/s11524-025-01001-x.

## Introduction

There is no official or standard definition of structural racism, but many racism scholars have highlighted its key tenets [1, 2]. Structural racism can operate without action at the individual level [2–4], interact with other forms of racism at multiple socioecological levels [4], and is reinforced by multiple systems of society [5]. A recent definition of structural racism accepted by many scholars is from Bailey et al.—“the totality of ways in which societies foster racial discrimination through mutually reinforcing inequitable systems (in housing, education, employment, earnings, benefits, credit, media, health care, criminal justice, and so on) that in turn reinforce discriminatory beliefs, values, and distribution of resources, which together affect the risk of adverse health outcomes [6] (p1454)”.

Past efforts have outlined the evolution of structural racism measurement methodology through to the current best practice [2, 7–11]. The current study fits into a set of studies that create multidimensional measures of structural racism using latent variable model approaches which are considered a best practice methodology [12–17]. Latent variable model approaches leverage manifest (i.e., observable or measurable) indicators that represent singular aspects of structural racism to estimate multidimensional structural racism as a latent variable (named latent because while a real construct, it cannot be directly measured). Their strength is in their ability to (1) capture the multidimensional and interactive qualities of structural racism even when there is correlation between the single indicators and (2) account for measurement error. Studies with such methodology to measure multidimensional structural racism have used either confirmatory factor analysis [12, 15–18] or latent class analysis (LCA) [13, 14]. The current study uses a method similar to LCA, called latent profile analysis (LPA) because the resultant categorical latent variable is more interpretable than a continuous one. Two recent studies used LPA to classify states into structural racism profiles [19, 20]. To date, the relevant studies have only measured structural racism at Public Use Microdata Areas in New York City (NYC) and Minnesota [13, 14] and across the U.S.—counties [12, 17], cities [15], and states [16, 18–20]. This study builds on this literature and is particularly a variation of the methodology in the Chantarat et al. papers that first used the term multidimensional measure of structural racism and the acronym MMSR for their latent class analyses at the Public Use Microdata Area geography [13, 14]. Given the limitations of prior research on measures of structural racism and the potential variation across geographies, this study expands on prior research in three ways. First, it improves upon existing measures of unidimensional structural racism indicators to yield a better multidimensional structural racism latent variable. Second, the study selected census tracts—the smallest geography used for structural racism measurement to date—because it is important to elucidate how structural racism, and its downstream impacts differ across and affect subcommunities. Since this study focuses on NYC, census tracts allow detection of patterns in a large, densely populated, and heterogeneous city to address structural racism and related adverse health outcomes more granularly than the citywide level. Third, this study sought to test the relationship between multidimensional measures of structural racism with community firearm violence in NYC. Community firearm violence—both fatal and nonfatal—harms population health, and it is crucial to understand the structural inequities that perpetuate violence [21, 22].

## Methods

### Sample

Census tracts in the United States generally have a population size of 1200–8000 with an ideal size of 4000. The NYC population is over 8 million; there are 2327 census tracts based on the 2020 U.S. Census for NYC. A total of 108 census tracts were excluded—41 uninhabitable (e.g., parks, airports, and cemeteries), 51 without either Black or non-Hispanic White residents, 15 with a population under 100, and Rikers Island prison. The final analytic dataset had 2219 NYC census tracts. The analytic census tract populations range from 109 to 17,222 (median of 3686; mean of 3964).

### Measures: Domains and Census Tract-level Indicators

Eight indicators across five domains (criminal justice, economic status/income, education, employment, and housing/segregation) were selected (Table 1; detailed indicator description in Appendix A; selection process in Appendix B). Most indicators—index of concentration at the extremes, low educational attainment, and unemployment—are from the 2018–2022 U.S. Census Bureau American Community Survey (ACS) 5-year estimates. The Index of Dissimilarity uses 2020 Decennial Census of Population and Housing population estimates. Drug-related arrests are from New York City Police Department (NYPD) Arrests Historic Dataset. The sociodemographic variables are from the 2018–2022 ACS (percent male, ages 15–44, living below 150% of the federal poverty level, and median household income) and 2020 Decennial Census (racial and ethnic composition and total population).

**Table 1:** Summary of unidimensional indicators of structural racism

Domain	Indicator name	Definition	Data source
Criminal justice	Drug-related arrests: Black and non-Hispanic White	Proportion of drug-related arrests among Black people	New York City Police Department (NYPD) Arrests Historic Data, 2018–2022
		Proportion of drug-related arrests among non-Hispanic White people	NYPD Arrests Historic Data, 2018–2022
Economic status/income	Index of concentration at the extremes (*ICE*) [23]	Racialized economic residential segregation: high racial privilege and high income versus low income and low racial privilege	U.S. Census Bureau American Community Survey (ACS), 2018–2022
Education	Low educational attainment: Black and non-Hispanic White	Proportion of Black adults ages 25 + without high school diploma	ACS 2018–2022
		Proportion of non-Hispanic White adults ages 25 + without high school diploma	ACS 2018–2022
Employment	Unemployment: Black and non-Hispanic White	Unemployment rate of Black civilians in the labor force ages 16–64 years	ACS 2018–2022
		Unemployment rate of non-Hispanic White civilians in the labor force ages 16–64 years	ACS 2018–2022
Housing/segregation	Index of Dissimilarity [14]	Racial residential segregation: Black versus non-Hispanic White *Conceptual*: measures the extent to which a region’s population would need to be redistributed across subunits to achieve a uniform distribution	2020 Decennial Census of Population and Housing

The 2018–2022 ACS did not publish data for non-Hispanic Black people as a separate category. Therefore, consistently across all unidimensional indicators, Black includes Hispanic and non-Hispanic ethnicity, while White is non-Hispanic only.

### Distal Outcome

The outcome is community firearm violence, defined here as the intentional, interpersonal use of a firearm that results in death or nonfatal injury [24]. To capture community firearm violence, we used the provided incident key to join the NYPD Shooting (contains location) and NYPD Shooting Victims (contains demographic variables) Datasets to measure the number of interpersonal fatal and nonfatal shootings by census tract from 2018–2022. The NYPD datasets exclude shootings caused by firearm suicide and suicide attempt and unintentional firearm injury and death. Since the focus is structural racism in the context of Black versus White social disadvantage, like the unidimensional indicators, the outcome is isolated to Black and non-Hispanic White people.

### Statistical Analyses

A latent profile analysis (LPA) with an expectation-maximum algorithm was conducted in Mplus to identify types of multidimensional structural racism, called profiles, among NYC census tracts (see Appendix C). The unit of analysis for this study is the census tract level, not person level. Using manifest (observed) unidimensional indicators of structural racism calculated for each census tract, the LPA detected latent (unobserved) profiles of structural racism that best group similar census tracts. In this study, the LPA relies on continuous unidimensional indicators of structural racism to capture the construct of multidimensional structural racism as a categorical latent variable. For the eight indicators, one- through seven- profile models were run to identify the best-fitting model. Multiple standard fit indices were compared [25].

In addition to fit statistics, classification quality with standardized entropy [26] and classification probabilities were considered. When the fit indices suggest more than one solution could be appropriate, substantive interpretation helps gauge if conceptually, the addition/exclusion of a profile has meaning because it is qualitatively different from existing profiles [25].The census tracts were assigned probabilities of membership for each possible profile, which helps account for measurement error. Exploratory data analysis was conducted to understand the distribution, missingness, and correlation between variables. All but 195 of the 2219 census tracts (8.78%) in the analytic dataset had data for all eight indicators. Data for those 195 census tracts were imputed in Mplus. By default, Mplus handles missing data with Full Information Maximum Likelihood estimation approach and assumes data are missing at random [27].

After selecting the best-fitting model, the shootings outcome and total population covariate (see Appendix D) were incorporated in the structural model using the maximum likelihood three-step approach by Vermunt [28]. A global Wald test and pairwise comparison *z*-tests were calculated to assess the difference in the shootings across the structural racism profiles, adjusted for total population. Shootings and structural racism profiles were mapped using the sf and ggplot2 packages in RStudio. Statistical analyses were conducted in Mplus Version 8.10 with the MplusAutomation R package. RStudio was also used for data management and visualizations. This study was deemed not human subjects research by the Johns Hopkins Bloomberg School of Public Health Institutional Review Board.

## Results

### Profile Enumeration

The five-profile model was selected (Table 2). The original latent profile model included all eight indicators with descriptive statistics shown in Appendix E. However, the mean Index of Dissimilarity (*DI*) value was nearly equivalent for all five profiles. As a result, one- through six- profile models were re-run without the *DI*, and the reported results only include the remaining seven indicators. There was a diminishing reduction in Bayesian Information Criteria (*BIC*) value when the fifth profile was added to the model. Additionally, the five-profile model split a larger profile of 1669 census tracts in the four-profile model into two profiles; these two profiles (later named Disadvantaged: Highest Education Inequity and Semi-Advantaged) had different mean *ICE* and Black drug-related arrests, which was substantively important to parse out. The smallest profile size in the five-profile model had 32 (1.4%) of the 2219 census tracts. Profiles containing less than 5–7% of the sample are considered small, but in this analysis, the small profile emerged in the three-profile model and persisted in the four-, five-, and six-profile models, which indicates it is a stable, actual profile. The entropy of 0.89 indicated the census tracts were sorted into their most probable profile with good certainty. In this study, the bootstrapped likelihood ratio test (*BLRT*) *p*-value did not help assess whether an additional profile led to a statistically significant improvement in the model fit [25].

**Table 2:** Fit indices and classification quality for latent 1- to 6-profile models

Number of								Smallest class size	
Classes	Parameters	Log likelihood	*AIC*	*BIC*	*VLMR p*-value	*BLRT p-*value	Entropy	*n*	%
1	14	5817.71	− 11,607.43	− 11,527.56	n/a	n/a	n/a	2219	100.00
2	22	6776.59	− 13,509.09	− 13,383.66	< 0.00005	0.002	0.93	421	19.00
3	30	7559.04	− 15,058.09	− 14,886.94	0.10	0.002	0.95	32	1.44
4	38	8219.98	− 16,363.96	− 16,147.18	0.01	0.002	0.95	32	1.44
***5***	***46***	***8721.50***	***− 17,350.99***	***− 17,088.57***	***0.02***	***0.002***	***0.89***	***32***	***1.44***
6	54	9190.99	− 18,273.98	− 17,965.92	0.23	0.002	0.89	32	1.44

Selected model in bold italic font.

*AIC* Akaike Information Criteria*, BIC* Bayesian Information Criteria, *BLRT* bootstrapped likelihood ratio test, *VLMR* Vuong-Lo-Mendell-Rubin adjusted likelihood ratio test, *n*/*a* not applicable

### Description of Structural Racism Profiles

The plot of the five-profile model means is in Appendix F. Each profile represents a multidimensional structural racism typology. The profiles were named by exploring the Black to non-Hispanic White inequity of within domain pair of indicator means for education, unemployment, and criminal justice (see Appendix G).

The first structural racism profile is the “Disadvantaged: Highest Criminal Justice Inequity” (*n* = 32, 1.4% of NYC census tracts). It was the smallest profile with the highest inequity in Black versus non-Hispanic White mean drug-related arrests. The *ICE* value was in the direction of deprivation; on average, 18% of the census tract was concentrated in the deprived group. The non-Hispanic White proportion unemployed had an outlier high mean. The Black drug-related arrests mean was over 22 times larger than the non-Hispanic White arrests.

Second is the “Disadvantaged: Highest Education Inequity” profile (*n* = 827, 37.3% of census tracts). This profile had the highest Black versus non-Hispanic White educational inequity. It had an *ICE* value near zero which signifies low racialized economic segregation. The mean Black unemployment was two times higher than that of non-Hispanic White people. The average proportion of Black drug-related arrests was highest, compared to the other profiles; the Black drug-related arrests mean was over 18 times larger than the non-Hispanic White arrests.

Third is “Disadvantaged: Highest Unemployment Inequity” (*n* = 104, 4.7% of census tracts). It is a small profile with the highest inequity in Black versus non-Hispanic White unemployment. The *ICE* value was in the direction of deprivation. This profile had the worst mean for Black and non-Hispanic White low educational attainment and Black unemployment.

The fourth profile is “Semi-Advantaged” (*n* = 882, 39.7% of census tracts). This profile aligns qualitatively with “lower” structural racism. The mean *ICE* value for the census tracts was − 0.26—on average, 26% of the census tract was concentrated into the privileged group. Aside from the economic status/income domain, the other domains have similar, but higher, Black versus non-Hispanic White values.

The last profile is “Advantaged” (*n* = 374, 16.9% of census tracts). It had the lowest *ICE*, in the privileged direction; on average, 28% of the census tract was in the privileged group. The mean for Black low educational attainment and Black unemployment were each similar but larger than the respective mean for non-Hispanic White people. The non-Hispanic White drug-related arrests mean was almost as high as the highest Black drug-related arrests mean across all profiles.

### Characteristics of Structural Racism Profiles

Using the most probable profile membership, the structural racism profiles were further characterized by various sociodemographic indicators (Table 3). The three Disadvantaged profiles had above average percent non-Hispanic Black people, below average median income, and above average poverty. The Semi-Advantaged profile had above average non-Hispanic White people; the Advantaged profile was majority non-Hispanic White. Both advantaged profiles had below average poverty.

**Table 3:** Mean and standard deviation of sociodemographic characteristics in New York City census tracts (*n* = 2219), citywide and by structural racism profile

	NYC		Disadvantaged: highest criminal justice inequity		Disadvantaged: highest education inequity		Disadvantaged: highest employment inequity		Semi-advantaged		Advantaged	
	2219 (100%)		32 (1.4%)		827 (37.3%)		104 (4.7%)		882 (39.7%)		374 (16.9%)	
	*Mean*	*sd*	*Mean*	*sd*	*Mean*	*sd*	*Mean*	*sd*	*Mean*	*sd*	*Mean*	*sd*
Total population (#)	3964	1927	3777	1385	4025	1914	3779	1693	4037	2109	3724	1559
Percent Asian, NH	15.7	17.4	8.0	17.0	7.0	10.0	9.6	14.4	23.5	19.9	19.0	15.5
Percent Black, NH	21.7	25.8	44.5	28.2	42.8	25.1	39.1	26.7	6.8	10.6	3.5	7.4
Percent Hispanic	26.9	21.1	39.1	27.5	31.8	23.1	39.2	25.8	25.5	19.1	14.7	9.2
Percent White, NH	30.4	27.4	2.1	1.7	12.4	16.9	5.4	12.4	39.1	24.8	58.8	20.0
Percent male	47.8	3.6	46.1	3.5	46.5	3.5	47.7	7.0	48.7	3.3	48.3	2.1
Percent ages 15–44	49.7	8.3	49.1	4.5	50.1	6.7	49.8	5.3	51.1	9.8	45.5	7.1
Percent below 150% FPL	24.9	15.3	34.2	17.8	30.8	16.4	35.2	17.9	20.3	12.2	19.2	11.5
Median income ($)	85,152	41,354	59,934	25,712	67,132	31,598	57,483	28,745	101,321	43,637	96,220	37,995

*NH* non-Hispanic; *sd* standard deviation; *FPL* federal poverty level

### Relationship between Structural Racism Profiles and Fatal and Nonfatal Community Firearm Violence

**Fig. 1:**
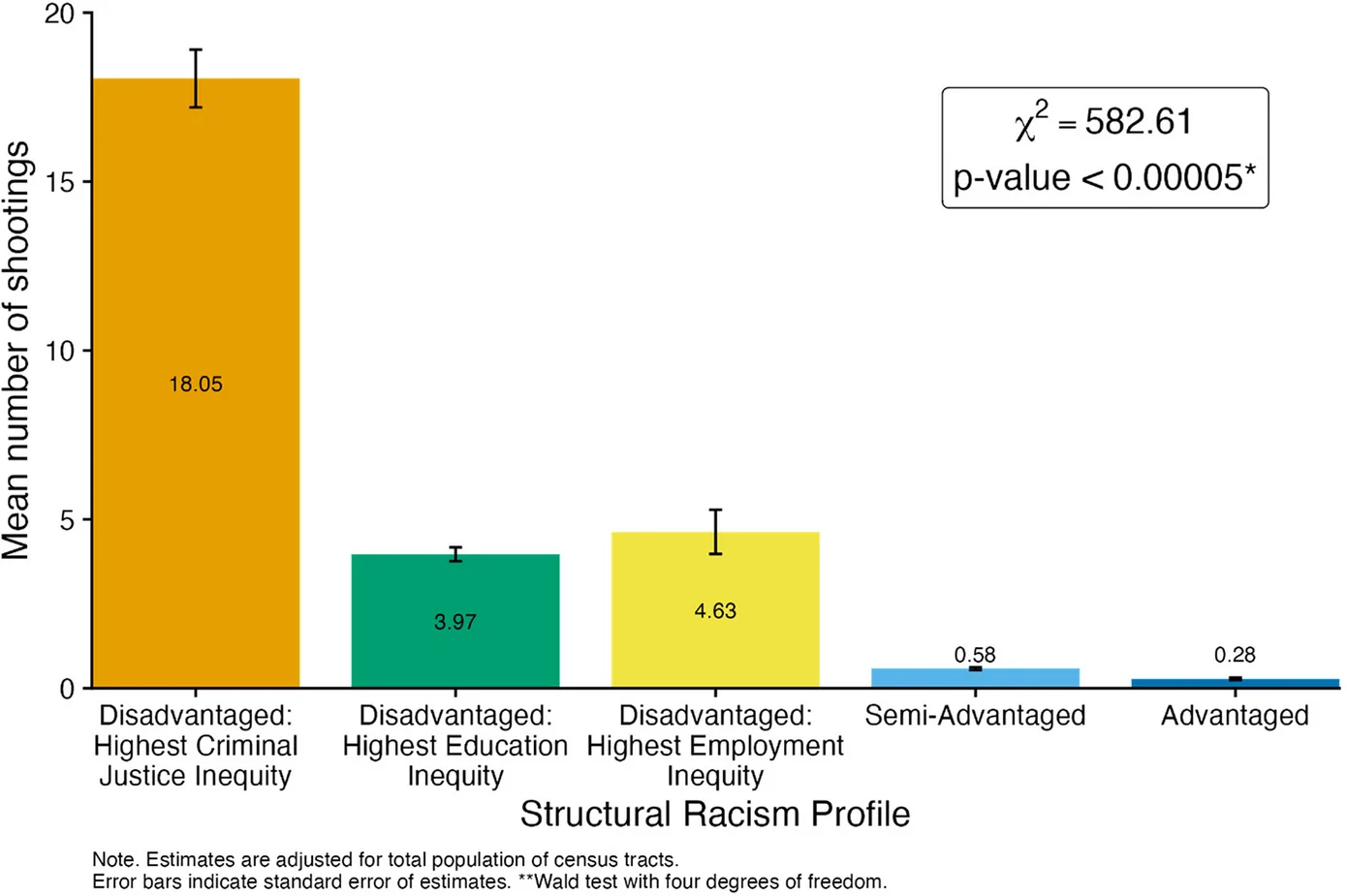
Prevalence of fatal and nonfatal shootings among Black and non-Hispanic White people in NYC by structural racism profile (2018–2022)

There were a total of 7015 shootings from 2018–2022 in the 2219 NYC census tracts; 5804 (82.7%) were among Black and non-Hispanic White people; 5654 of the 5804 shootings (97.4%) were among Black people. The global Wald test showed the mean number of shootings among Black and non-Hispanic White people significantly differed across the structural racism profiles. The Wald chi-squared value was 582.61; and *p*-value was < 0.00005 (Fig. 1).

Overall, the census tracts categorized as Disadvantaged structural racism profiles had higher prevalence of shootings than the Advantaged and Semi-Advantaged profiles. The Disadvantaged: Highest Criminal Justice Inequity profile had the highest mean number of shootings—18. The Disadvantaged: Highest Education Inequity and Disadvantaged: Highest Employment Inequity profiles had similar means of 4.0 and 4.6, respectively. The Semi-Advantaged and Advantaged profiles had low mean number of shootings (0.6 and 0.3, respectively).

The pairwise comparison *z*-tests of number of shootings across structural racism profiles, adjusted for total population, are in Appendix H. All but one pairwise comparison (Disadvantaged: Highest Education Inequity versus Disadvantaged: Highest Employment Inequity) was statistically significant.

**Fig. 2:**
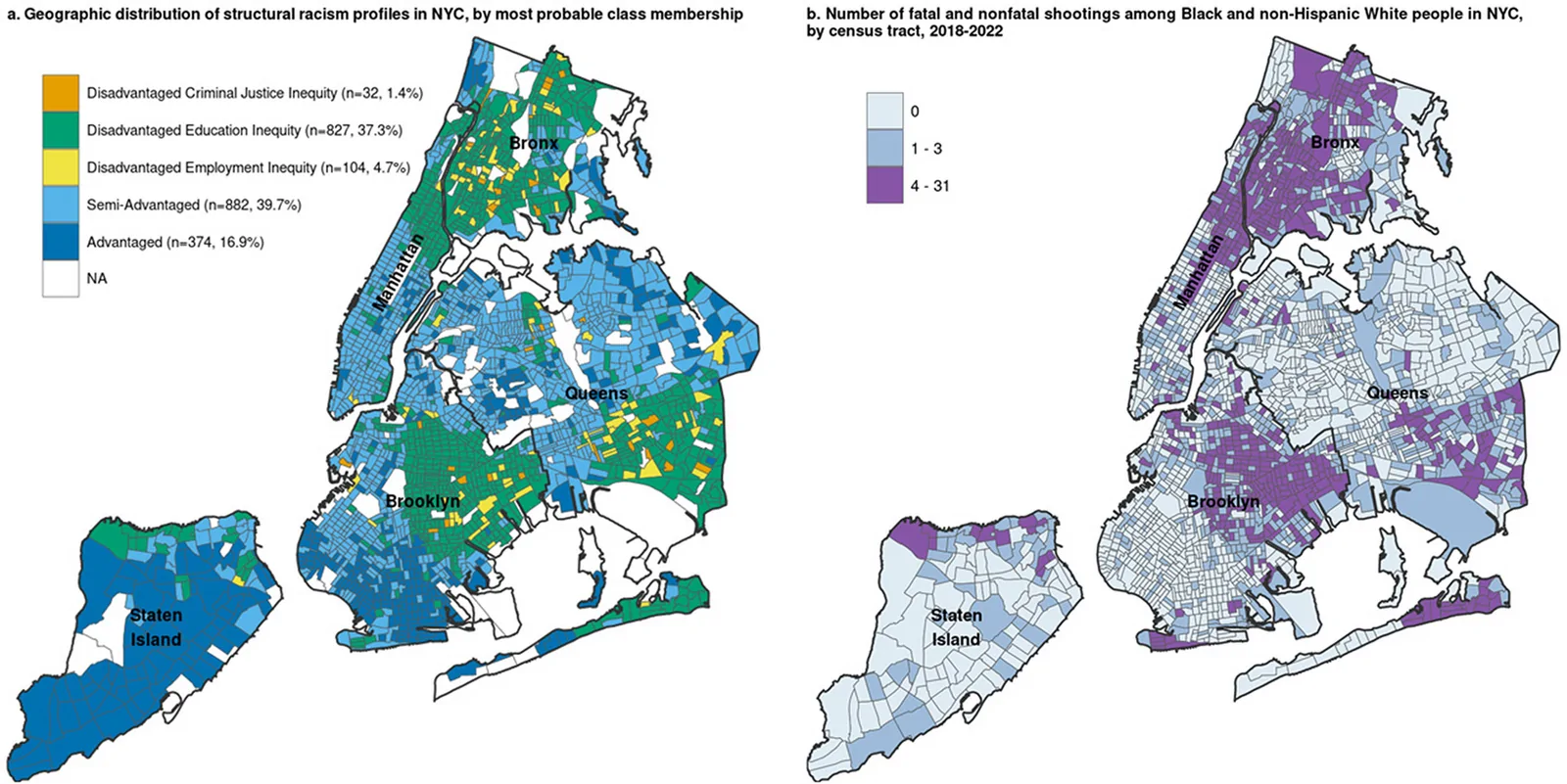
Geographic distribution of structural racism profiles and shootings. In Fig 2a, the white areas on the map (NA in the legend) are the 108 NYC census tracts excluded from the latent profile analysis. Data source for Fig. 2b: NYPD Shootings and Shooting Victims Datasets, 2018–2022; data represent fatal and nonfatal shootings combined. Median cutpoint of 3 was calculated among all NYC census tracts (*n* = 2327) with non-zero shootings

Figure 2 visualizes the geographic distribution of the five structural racism profiles and shootings, side by side. In Fig. 2a, light and dark blue are the advantaged profiles. Census tracts in the advantaged profiles are close to one another, especially in most of Staten Island, Midtown and Lower Manhattan, the Southwestern and Western parts of Brooklyn, and all but the Southeastern parts of Queens. Green represents the largest of the disadvantaged profiles—this profile is located in Northern Manhattan and into the Bronx, Eastern Brooklyn, and Southeastern Queens. Figure 2b illustrates the number of shootings among Black and non-Hispanic White people between 2018–2022; dark purple is above median shootings. The maps generally show that higher prevalence shootings and the Disadvantaged structural racism profiles co-occur in the same census tracts and areas, while the Advantaged and Semi-Advantaged have lower shootings.

## Discussion

To our knowledge, this is the first paper to use latent profile analysis to create a multidimensional measure of structural racism (MMSR) specifically at the census tract level—the smallest level of geography used in similar papers. Based on seven indicators used to create the MMSR, we identified five meaningful multidimensional structural racism profiles in NYC that highlight important inequities in the interconnected domains of criminal justice, economic status/income, education, employment, and housing/segregation. The index of dissimilarity (DI) measures the distribution of groups within a geographic unit by looking at subunits (e.g., comparing distribution of Black and non-Hispanic White people in census block subunits, compared to the larger census tract unit). *ICE* instead of DI was the sole indicator in the housing/segregation domain because the latter was identical for all profiles [29]. This aligns with prior work that explains that the index of dissimilarity is less useful at smaller geographies like census tracts because people tend to be more alike; the DI is more commonly used as a segregation measure for city level and larger [29, 30]. These results underscored that the final seven indicators were of sufficient quality because they separated the census tracts into profiles with substantive meaning. What stood out most about the five structural racism profiles is that two had “less” structural racism in relation to the other three profiles. Nominally, these were labeled Semi-Advantaged and Advantaged versus three Disadvantaged profiles.

This study also explored the utility of the MMSR by investigating if the structural racism profiles were associated with community firearm violence. The prevalence of shootings differed significantly across the structural racism profiles. Census tracts in the three disadvantaged profiles experienced more shootings on average than those in the two advantaged profiles. Structural racism generates racialized health inequities. The literature demonstrates that community firearm violence is tied to various social and structural determinants such as—concentrated poverty and low socioeconomic status, neighborhood vacancy and blight, and lack of resources to meet needs and allow for social mobility—that are conducive to violence over safety in Black communities [22, 30–35].

There are at least seven studies that measured structural racism as a latent construct, and they generally showed important impacts of structural racism on health [12–20]. Of particular relevance are the three studies by Siegel et al. at the county, city, and state levels because they found an association between their structural racism measure (developed with confirmatory factor analysis) and Black-White firearm homicide disparities [15–17]. Every one standard deviation increase in the structural racism factor score was associated with an increase in the Black-White firearm homicide ratio—the magnitude of this increase was 1.6 at the county-level, 1.2 at the city-level, and 4.5 at the state-level [15–17]. The findings of the current study align with the aforementioned literature by showing that yet another public health outcome—community firearm violence (inclusive of nonfatal shootings)—is associated with structural racism. Future research should explore the exact mechanisms by which structural racism is on the pathway to community firearm violence.

The sociodemographic characteristics of each profile revealed important context. The two advantaged structural racism profiles had above the NYC average percentage of non-Hispanic White residents, while the three disadvantaged profiles had above average non-Hispanic Black residents. The inequities in the various domains used to create the MMSR are not due to the mere existence of Black people in the census tracts; this misconception would mean that to improve public health, Black people would have to move to neighborhoods without Black people [13]. This is flawed—the MMSR deflects away from problematizing individual-level, socially constructed factors like race to instead emphasizing the need to improve the entire system of structural racism from a lens of upstream root causes of social injustice.

Looking at the geographic distribution, there is a prominent similarity in the census tracts most negatively impacted by structural racism (i.e., the disadvantaged profiles from the MMSR) and shootings. Since this research improves upon studying these social determinants of health individually and instead combines these dimensions into an MMSR, it is unsurprising that shootings and the disadvantaged structural racism profiles co-exist. This highlights that Black people are exposed to environments with higher disadvantage, as measured by the MMSR and higher shootings.

### Limitations

The results of this study should be interpreted considering a few limitations. The first set of limitations relates to census tract geography. The modifiable areal unit problem (MAUP) describes how aggregation to different spatial units can modify findings because the units are somewhat arbitrary [36]. Because of MAUP and otherwise, there is no consensus on the best geography to measure structural racism. Some scholars argue that census tracts cannot easily be connected to policies that improve health inequities because policies that have institutionalized structural racism are made at larger geographies [37]. This fails to recognize that quantifying structural racism profiles by census tract still informs policy development, interventions, and resource allocation to reach subcommunities most impacted. Policies, laws, programs, and practices that perpetuate structural racism can operate at federal, state, and local levels; their effects are felt at different levels too (i.e., a state policy could differentially impact a small subset of the state). Future work should explore what strategies can prevent firearm violence by improving drivers of structural racism at various levels (e.g., city, state, and federal) that have disproportionate negative impacts on specific places and communities in NYC.

The ACS census tract estimates can have large margins of error (*MOE*) because tracts are relatively small [38]. To mitigate this concern, we used ACS 5- instead of 1-year estimates which have smaller *MOE *[38]. This also meant that for some variables there were small census tracts with responses by few to no Black or non-Hispanic White people. This led to some missing data that were imputed in Mplus (only 9% of census tracts had at least one imputed variable). However, the benefits of granular patterns still outweigh measuring structural racism citywide in NYC. Also, interpersonal shootings affect hyperlocal areas of cities, so census tract geography is important for identifying community firearm violence drivers.

The study does not directly account for multidimensional structural racism overtime because the MMSR represents structural racism in NYC census tracts at one-time period. But, since the effects of structural racism are lagged, persistent, and accumulating, the cross-sectional MMSR may encompass the ripple effects of deeply entrenched structural racism and resulting disadvantage from years of policies and practices that disadvantage Black communities [1, 6]. The aim was to develop a strong MMSR for census tracts at one-time point; as mentioned by Chantarat et al. and explored by Uzzi et al. for residential segregation specifically, future work can track structural racism over multiple time periods [13, 34] and account for historical structural racism [20, 39].

Although commonly used and relatively complete, it is possible that police data do not include shootings known to hospitals, trauma registries, or completely unknown/unreported [40]. The NYPD classifies race and ethnicity of people injured by firearms which can introduce bias and misclassification.

Finally, since the MMSR was developed for densely populated NYC, this study’s methods and findings might not be generalizable to other metropolitan areas or reproducible in less populated areas.

## Conclusion

These findings suggest that there is a benefit of operationalizing structural racism as a multidimensional construct. The chosen combination of unidimensional indicators can approximate profiles of structural racism that help predict higher versus lower shootings. This research builds on pioneering structural racism measurement scholarship to add a replicable, theory-informed, and empirical MMSR at the census tract level. Given that many NYC census tracts are impacted by both structural racism and shootings—the most sustainable solutions to advance health equity will be targeted, place-based solutions that invest in improving multiple social and structural determinants to prevent violence in these most impacted communities.

## Supplementary Information

Below is the link to the electronic supplementary material.Supplementary file1 (DOCX 338 KB)

## Data Availability

The data used in this study are publicly available. Additional details about the unidimensional indicators are provided in the Electronic Supplementary Materials. The U.S. Census Bureau American Community Survey (ACS): https://www.census.gov/data/developers/data-sets/acs-5year.html Decennial Census of Population and Housing: https://www.census.gov/programs-surveys/decennial-census.html New York City Police Department (NYPD) Arrests Historic Data: https://data.cityofnewyork.us/Public-Safety/NYPD-Arrests-Data-Historic-/8h9b-rp9u/about_data NYPD Shootings (2006-Present): https://data.cityofnewyork.us/d/5ucz-vwe8 NYPD Shooting Victims (2006-Present): https://data.cityofnewyork.us/d/pztn-9bne

## Abbreviations

NYC: New York City
LCA: Latent class analysis
LPA: Latent profile analysis
NYPD: New York City Police Department
ACS: American Community Survey
*ICE*: Index of Concentration at the Extremes
*DI*: Index of Dissimilarity
*AIC*: Akaike Information Criteria
*BIC*: Bayesian Information Criteria
*BLRT*: Bootstrapped likelihood ratio test
*VLMR*: Vuong-Lo-Mendell-Rubin adjusted likelihood ratio test
NH: Non-Hispanic
*sd*: Standard deviation
FPL: Federal poverty level
MAUP: Modifiable areal unit problem
MMSR: Multidimensional measure of structural racism
*MOE*: Margins of error
