# Supplementary material for: A Multidimensional Approach to Measure Structural Racism and Its Association with Fatal and Nonfatal Community Firearm Violence at the Census Tract Level in New York City
Source: J Urban Health. 2026 Jun 22;103(4):628–38. doi: 10.1007/s11524-025-01001-x (PMC13524899; doi:10.1007/s11524-025-01001-x)
Supplement: Supplementary file 1 [file 11524_2025_1001_MOESM1_ESM.docx]

**Appendices**

I. Appendix A: Latent profile indicators, conceptual meaning, computation, and data sources, by domain
II. Appendix B: Indicator selection process
III. Appendix C: Diagram of latent profile model
IV. Appendix D: Description of covariate selection
V. Appendix E: Descriptive characteristics of the unidimensional structural racism indicators for NYC and by latent profile for the model with Index of Dissimilarity
VI. Appendix F: Mean of unidimensional indicators of structural racism for census tracts in each latent profile in the 5-profile model
VII. Appendix G: Description of how profiles were named
VIII. Appendix H: Pairwise comparisons of the mean number of fatal and nonfatal shootings across the five structural racism profiles
IX. References for Electronic Supplemental Material

***Appendix A: Latent profile indicators, conceptual meaning, computation, and data sources, by domain***

| **Domain** | **Indicator name** | **Conceptual meaning** | **Computation** | **Data source** | **Variables** | **Status** |
| --- | --- | --- | --- | --- | --- | --- |
| Housing/  Segregation | Index of Dissimilarity (DI) | Racial residential segregation: Black v non-Hispanic White  *Conceptual*: measures the extent to which a region’s population would need to be redistributed across subunits to achieve a uniform distribution. | $D = \frac{1}{2}\sum_{i=1}^{n} \left\vert\left( \frac{w_{i}}{w_{T}}-\frac{b_{i}}{b_{T}} \right) \right\vert$  where b = N of Black persons in smaller areal unit and B = N of Black persons in the larger areal unit; w and W are corresponding values for the non-Hispanic White population. In our study, we used the census block group as the smaller areal unit and census tract as the larger areal unit.  The index ranges 0 to 1. High values indicate the two groups share no sub-unit areas in common, and low values indicate that all sub-unit areas include the same relative numbers of the two groups included in the larger area.^1^ | [Decennial Census of Population and Housing](https://www.census.gov/programs-surveys/decennial-census/data.html), 2020 | P1_004N  P2_005N | Dropped from model |
| Education | Low educational attainment | Proportion of Black adults ages 25+ without high school diploma | Numerator: Number of Black people ages 25 and over without a high school diploma  Denominator: Number of Black people ages 25 and over | [U.S. Census Bureau American Community Survey (ACS)](https://www.census.gov/data/developers/data-sets/acs-5year.html), 2018-2022 | Numerator: C15002B_003E +  C15002B_008E  Denominator:  C15002B_001E | Included in model |
|  |  | Proportion of non-Hispanic White adults ages 25+ without high school diploma | Numerator: Number of non-Hispanic White people ages 25 and over without a high school diploma  Denominator: Number of non-Hispanic White people ages 25 and over | ACS, 2018-2022 | Numerator:  C15002H_003E + C15002H_008E  Denominator:  C15002H_001E | Included in model |
| Employment | Unemployment | Unemployment rate of Black civilians in the labor force ages 16-64 years | Numerator: Number unemployed Black people ages 16-64 years in the civilian labor force  Denominator: Number of Black people ages 16-64 years in the civilian labor force | ACS, 2018-2022 | Numerator:  C23002B_008E + C23002B_021E  Denominator:  C23002B_001E | Included in model |
|  |  | Unemployment rate of non-Hispanic civilians in the labor force ages 16-64 years | Number unemployed non-Hispanic White people ages 16-64 years in the civilian labor force  Denominator: Number of non-Hispanic White people ages 16-64 years in the civilian labor force | ACS, 2018-2022 | Numerator:  C23002H_008E + C23002H_021E  Denominator:  C23002H_001E | Included in model |
| Criminal Justice | Drug-related arrests | Proportion of drug-related arrests among Black people | Numerator: Number of Black people arrested for drug-related crime  Denominator: Total number of drug-related arrests | [New York City Police Department (NYPD) Arrests Historic Data](https://data.cityofnewyork.us/Public-Safety/NYPD-Arrests-Data-Historic-/8h9b-rp9u/about_data), 2018-2022 | NYPD internal classification codes (KY_CD variable in dataset) related to drugs – 117, 235, 250, 365 | Included in model |
|  |  | Proportion of drug-related arrests among non-Hispanic White people | Numerator: Number of non-Hispanic White people arrested for drug-related crime  Denominator: Total number of drug-related arrests | NYPD Arrests Historic Data, 2018-2022 | NYPD internal classification codes (KY_CD variable in dataset) related to drugs – 117, 235, 250, 365 | Included in model |
| Economic status/Income | Index of concentration at the extremes (ICE) | Racialized economic residential segregation: high racial privilege + high income vs. low income and low racial privilege | $ICE =\frac{D -P}{T}$  Where D = number of Black households with income lower than $30,000 (deprived group);  P = number of non-Hispanic White households with income of $150,000 or higher (privileged group);  T = total number of Black and non-Hispanic White households in the census tract.  This index ranges from -1 (all households are in the privileged group) to 1 (all households are in the deprived group)*.^2^  *flipped from common calculation, to orient where bigger number means worse outcomes for Black people | ACS, 2018-2022 | *D*: B19001B_002E + B19001B_003E + B19001B_004E + B19001B_005E + B19001B_006E  *P*:  B19001H_016E + B19001H_017E  *T*:  B19001B_001E +  B19001H_001E | Included in model |

***Appendix B: Indicator selection process***

The unidimensional indicators had to meet the following criteria:

(1) Capture one of the nine domains in the Bailey et al.^3^ structural racism definition—housing, education, employment, earnings, benefits, credit, media, health care, and criminal justice,

(2) Preferably used in a previous study,

(3) Publicly available from U.S. Census Bureau surveys or other open data sources,

(4) Available at the census tract level, and

(5) Stratified by race and ethnicity.

First, indicators from previous studies that aimed to measure multidimensional structural racism were reviewed, especially those that employed similar mixture modeling methods as the current study.^4–10^ In some cases, the variables were unsuitable because they are not available for census tracts and it was not preferable to assign data from a larger geography (e.g., county) to census tracts. This study also relied on existing systematic reviews with a list of structural racism measures^4–6^ and literature on social and structural determinants of health.^7–11^ Finally, the ACS was reviewed for variables that were not discussed in the selected reviewed literature; the ACS review did not yield additional variables. The goal was to include indicators from as many of the Bailey et al. nine domains as possible, but variables that met all the criteria were not identified for the domains of benefits and credit.^3^ Instead, the study includes related variables that capture other aspects of socioeconomic status like income and employment. A structural racism indicator in the health care domain that met all the criteria was not identified. In the variable selection process, domains used in other studies beyond the Bailey et al. domains were considered like built environment, environmental racism, political participation, social cohesion, and transportation. However, this study ultimately focused on a parsimonious set of indicators most aligned with the domains in the Bailey et al. definition and used in the majority of relevant studies.^3^

***Appendix C: Diagram of latent profile model***

***Appendix D: Description of covariate selection***

We included one covariate—total population at the census tract level, from the 2020 Decennial Census. This variable was included because while census tracts are intended to be a similar size of 1,200 to 8,000, there are some outliers. Only 107 out of 2,219 (4.8%) census tracts in the analytic dataset have populations outside this range. Since the total population might predict or influence the structural racism latent variable and total population (and density) are also shown to be related to the outcome of firearm violence, it was adjusted for in these analyses.^12^ The total population was split into three categories with population size of (1) less than 1,200, (2) 1,200 to 8,000, and (3) greater than 8,000. The total population categories were included in the model as dummy variables, with the 1,200 to 8,000 category as the reference group.

Previous studies that employed mixture modeling methods to measure structural racism and then test the association with a health outcome controlled for covariates (e.g., high school degree, unemployment, poverty). However, many of these commonly used variables are already represented in the MMSR.^12,13^

***Appendix E: Descriptive characteristics of the unidimensional structural racism indicators for NYC and by latent profile for the model with Index of Dissimilarity^1^***

| Unidimensional indicator | NYC | | Disadvantaged: Highest Criminal Justice Inequity | | Disadvantaged: Highest Education Inequity | | Disadvantaged: Highest Employment Inequity | | Semi-Advantaged | | Advantaged | |
| --- | --- | --- | --- | --- | --- | --- | --- | --- | --- | --- | --- | --- |
|  | 2219 (100%) | | 32 (1.4%) | | 827 (37.3%) | | 104 (4.7%) | | 881 (39.7%) | | 375 (16.9%) | |
|  | N  (%) | mean (sd) | N  (%) | mean (sd) | N  (%) | mean (sd) | N  (%) | mean (sd) | N  (%) | mean (sd) | N  (%) | mean (sd) |
| Index of Concentration at the Extremes | 2210 (99.6%) | -0.10 (0.26) | 31 (96.9%) | 0.18  (0.15) | 825 (99.8%) | 0.09 (0.18) | 102 (98.1%) | 0.19 (0.17) | 877 (99.5%) | -0.26 (0.20) | 375 (100%) | -0.28 (0.15) |
| Drug-related arrests: Black | 2219 (100%) | 0.43 (0.33) | 32 (100%) | 0.68 (0.28) | 827 (100%) | 0.74 (0.19) | 104 (100%) | 0.62 (0.26) | 881 (100%) | 0.26 (0.22) | 375 (100%) | 0.10 (0.14) |
| Drug-related arrests: White, NH | 2219 (100%) | 0.20 (0.26) | 32 (100%) | 0.03 (0.04) | 827 (100%) | 0.04 (0.06) | 104 (100%) | 0.06 (0.09) | 881 (100%) | 0.15 (0.14) | 375 (100%) | 0.69 (0.18) |
| Low educational attainment: Black | 2079 (93.7%) | 0.15 (0.17) | 32 (100%) | 0.18 (0.19) | 820 (99.2%) | 0.16 (0.11) | 102 (98.1%) | 0.19 (0.13) | 811 (92.1%) | 0.13 (0.19) | 314 (83.7%) | 0.14 (0.21) |
| Low educational attainment: White, NH | 2167 (97.7%) | 0.09  (0.14) | 32 (100%) | 0.12 (0.26) | 782 (94.6%) | 0.06  (0.09) | 103 (99.0%) | 0.59 (0.21) | 875 (99.3%) | 0.06 (0.07) | 375 (100%) | 0.08 (0.06) |
| Unemployment: Black | 2093 (94.3%) | 0.05 (0.09) | 32 (100%) | 0.06  (0.06) | 822 (99.4%) | 0.06 (0.06) | 103 (99.0%) | 0.07 (0.06) | 820 (93.1%) | 0.04 (0.09) | 316 (84.3%) | 0.04 (0.12) |
| Unemployment: White, NH | 2174 (98.0%) | 0.04 (0.07) | 32 (100%) | 0.47 (0.20) | 786 (95.0%) | 0.03 (0.05) | 104 (100%) | 0.02 (0.04) | 877 (99.5%) | 0.03 (0.04) | 375 (100%) | 0.03 (0.03) |
| Index of Dissimilarity | 2218 (100%) | 0.18 (0.15) | 32 (100%) | 0.17 (0.13) | 827 (100%) | 0.18 (0.15) | 104 (100%) | 0.17 (0.13) | 880 (99.9%) | 0.18 (0.15) | 375 (100%) | 0.18 (0.14) |

Notes: NYC = New York City; sd = standard deviation; NH = Non-Hispanic. There are instances in the table where the percentage rounds up to 100.

^1^The Index of Dissimilarity was removed in the final selected model.

^2^The selected five-profile model with seven indicators (Index of Dissimilarity was removed) has 882 census tracts in the Semi-Advantaged profile and 374 census tracts in the Advantaged profile (instead of 881 and 375, respectively, seen in Appendix E table). However, the mean and standard deviation for the selected model were identical for NYC and each profile, so a separate table is not presented. The means from the selected model are visualized in the plot in Appendix F.

***Appendix F: Mean of unidimensional indicators of structural racism for census tracts in each latent profile in the 5-profile model***

***Appendix G: Description of how profiles were named***

The profiles were named by exploring the Black to non-Hispanic White inequity of within domain pair of indicator means for education, unemployment, and criminal justice.

For instance, for the education domain, the mean Black low educational attainment (measured as the proportion of Black adults ages 25+ without high school diploma) was compared to the mean non-Hispanic White low educational attainment as a ratio, to understand the within-domain indicator relationships. This procedure was followed for domain pairs in each profile.

The profile name highlights a unique feature (i.e., single domain) but given that this is a multidimensional measure of structural racism, there are other important inequities and patterns across the indicator means within and across profiles.

The fourth and fifth profiles (Semi-Advantaged and Advantaged) have “lower” structural racism and social disadvantage and thus have Advantaged in their name. Those two profiles are more similar to each other and different from the other three profiles that have “higher” structural racism and social disadvantage and thus those three have Disadvantaged in their name.

***Appendix H: Pairwise comparisons of the mean number of fatal and nonfatal shootings across the five structural racism profiles***

| **Pairwise Comparison** | **Prevalence Difference** | **S.E.** | **p-value** |
| --- | --- | --- | --- |
| Disadvantaged: Highest Education Inequity vs.  Semi-Advantaged | 3.39 | 0.21 | <0.0005 |
| Disadvantaged: Highest Education Inequity vs.  Disadvantaged: Highest Employment Inequity | -0.66 | 0.72 | 0.36 |
| Disadvantaged: Highest Education Inequity vs.  Disadvantaged: Highest Criminal Justice Inequity | -14.08 | 0.77 | <0.0005 |
| Disadvantaged: Highest Education Inequity vs.  Advantaged | 3.69 | 0.21 | <0.0005 |
| Semi-Advantaged vs.  Disadvantaged: Highest Employment Inequity | -4.05 | 0.65 | <0.0005 |
| Semi-Advantaged vs.  Disadvantaged: Highest Criminal Justice Inequity | -17.47 | 0.85 | <0.0005 |
| Semi-Advantaged vs.  Advantaged | 0.30 | 0.05 | <0.0005 |
| Disadvantaged: Highest Employment Inequity vs.  Disadvantaged: Highest Criminal Justice Inequity | -13.42 | 1.07 | <0.0005 |
| Disadvantaged: Highest Employment Inequity vs.  Advantaged | 4.35 | 0.65 | <0.0005 |
| Disadvantaged: Highest Criminal Justice Inequity vs.  Advantaged | 17.77 | 0.85 | <0.0005 |

*Note. S.E. = standard error*

**References**

1. Chantarat T, Van Riper DC, Hardeman RR. Multidimensional structural racism predicts birth outcomes for Black and White Minnesotans. *Health Serv Res*. 2022;57(3):448-457. doi:10.1111/1475-6773.13976
2. Krieger N, Waterman PD, Spasojevic J, Li W, Maduro G, Van Wye G. Public Health Monitoring of Privilege and Deprivation With the Index of Concentration at the Extremes. *Am J Public Health*. 2016;106(2):256-263. doi:10.2105/AJPH.2015.302955
3. Bailey ZD, Krieger N, Agénor M, Graves J, Linos N, Bassett MT. Structural racism and health inequities in the USA: evidence and interventions. *Lancet*. 2017;389(10077):1453-1463. doi:10.1016/S0140-6736(17)30569-X
4. Wien S, Miller AL, Kramer MR. Structural racism theory, measurement, and methods: A scoping review. *Front Public Health*. 2023;11:1069476. Published 2023 Feb 16. doi:10.3389/fpubh.2023.1069476
5. Hing AK, Chantarat T, Fashaw-Walters S, Hunt SL, Hardeman RR. Instruments for racial health equity: a scoping review of structural racism measurement, 2019-2021. *Epidemiol Rev*. 2024;46(1):1-26. doi:10.1093/epirev/mxae002
6. Groos M, Wallace M, Hardeman R. Measuring inequity: a systematic review of methods used to quantity structural racism. J Health Dispar Res Pract. 2018;11(2):13. Accessed March 27, 2023. https://digitalscholarship.unlv.edu/jhdrp/vol11/iss2/13
7. Riley AR. Neighborhood Disadvantage, Residential Segregation, and Beyond-Lessons for Studying Structural Racism and Health. *J Racial Ethn Health Disparities*. 2018;5(2):357-365. doi:10.1007/s40615-017-0378-5
8. Lukachko A, Hatzenbuehler ML, Keyes KM. Structural racism and myocardial infarction in the United States. *Soc Sci Med*. 2014;103:42-50. doi:10.1016/j.socscimed.2013.07.021
9. Wallace M, Crear-Perry J, Richardson L, Tarver M, Theall K. Separate and unequal: Structural racism and infant mortality in the US. *Health Place*. 2017;45:140-144. doi:10.1016/j.healthplace.2017.03.012
10. Bell CN, Kerr J, Young JL. Associations between Obesity, Obesogenic Environments, and Structural Racism Vary by County-Level Racial Composition. *Int J Environ Res Public Health*. 2019;16(5):861. Published 2019 Mar 9. doi:10.3390/ijerph16050861
11. Chambers BD, Erausquin JT, Tanner AE, Nichols TR, Brown-Jeffy S. Testing the Association Between Traditional and Novel Indicators of County-Level Structural Racism and Birth Outcomes among Black and White Women. *J Racial Ethn Health Disparities*. 2018;5(5):966-977. doi:10.1007/s40615-017-0444-z
12. Siegel M, Rieders M, Rieders H, et al. Measuring Structural Racism and Its Association with Racial Disparities in Firearm Homicide. *J Racial Ethn Health Disparities*. 2023;10(6):3115-3130. doi:10.1007/s40615-022-01485-2
13. Dougherty GB, Golden SH, Gross AL, Colantuoni E, Dean LT. Measuring Structural Racism and Its Association With BMI. *Am J Prev Med*. 2020;59(4):530-537. doi:10.1016/j.amepre.2020.05.019
